# Scott-Taor Syndrome: A Clinical Case Report

**DOI:** 10.7759/cureus.51437

**Published:** 2024-01-01

**Authors:** Rui L Madureira, Adélio Vilaça, Paulo Pereira, Teresa Gomes, Xénia Verraest

**Affiliations:** 1 Physical Medicine and Rehabilitation, Centro Hospitalar Universitário do Algarve, Faro, PRT; 2 Orthopaedics, Centro Hospitalar Universitário de Santo António, Porto, PRT; 3 Physical Medicine and Rehabilitation, Centro de Medicina de Reabilitação da Região Centro-Rovisco Pais, Coimbra, PRT

**Keywords:** tbx4 gene, coxopodopatellar syndrome, small patella syndrome, ischiopatellar dysplasia, scott-taor syndrome

## Abstract

Scott-Taor syndrome is a benign bone dysplasia with less than 50 cases reported. The latter is an autosomal dominant disease characterized mainly by patellar a/hypoplasia and bilateral malunion ossification of the ischiopubic junction, a wide gap between the first and second toes. The diagnosis is clinical and radiographical. Here, we present a 17-year-old female patient with this rare syndrome. Considering this diagnosis is crucial for a better understanding of the pathology and to effectively contextualize the patient's clinical findings.

## Introduction

Scott-Taor syndrome, also known as ischiopatellar dysplasia, small patella syndrome, or coxopodopatellar syndrome, is a rare benign bone dysplasia with less than 50 cases reported [1]. The latter is an autosomal dominant disease caused by mutations of the human TBX4 gene (chromosome 17q22) [2]. The main clinical features include patellar a/hypoplasia and bilateral absent, delayed, or irregular ossification of the ischiopubic junction and the infra-acetabular axe cut notches [3]. Other major signs are a wide gap between the first and second toes, short fourth and fifth rays of the feet, and pes planus. Less commonly, elongated femoral necks, flattened and widened proximal femoral epiphyses, hypoplasia of the lesser trochanter, and tarsal anomalies can be present [4]. Intrafamilial variability of the patellar, pelvic, and foot anomalies has been described, and it should be clinically differentiated from disorders with a/hypoplastic patellae, like familial patella aplasia-hypoplasia syndrome [5], and nail-patella syndrome [6]. 

The presentation varies from asymptomatic to knee pain, knee osteoarthritis in elderly subjects, or recurrent luxations from infancy. The diagnosis is clinical and radiographical [4].

## Case presentation

A 17-year-old female, born to non-consanguineous parents, exhibited typical development. She presented to our hospital's orthopedic department with a history of psoriasis since the age of eight, managed with topical treatment. The primary complaint was mechanical pain associated with a sensation of patellar instability, mostly during physical education lessons, persisting for the last two years, without documented dislocation episodes. No additional musculoskeletal issues were reported.

**Figure 1:**
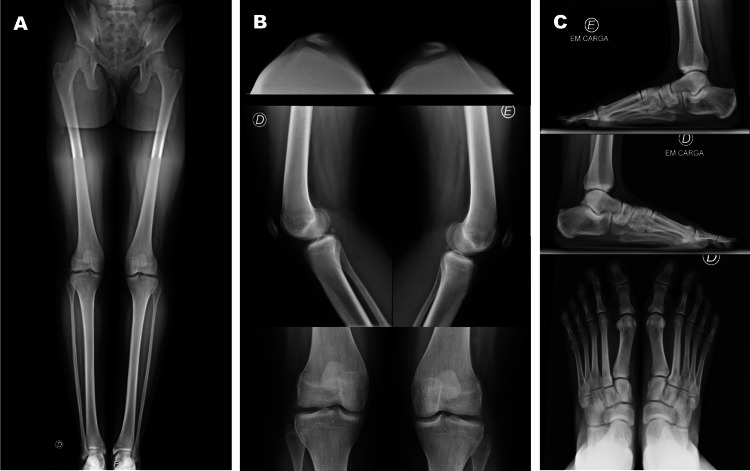
X-rays of the patient: (A) delayed ossification of the ischiopubic junction. (B) bilateral patellar hypoplasia. (C) reduced longitudinal arches of the feet and widened gap between the first and second toes.

Clinical examination revealed bilateral hypoplastic patellae, positive apprehension on testing, an inability to perform a monopodal squat because of muscular weakness and pain, and a pressure callus on the sole combined with reduced longitudinal arches of the feet. The radiographic analysis demonstrated an absent ischiopubic junction, bilateral patellar hypoplasia, and reduced longitudinal arches of the feet (Figure 1).

Family history revealed the patient's father who is a 49-year-old and who also sought orthopedic consultation for left knee pain because of osteoarthritis and bilateral patellar hypoplasia. The patient was awaiting assessment by vascular surgery because of marked venous alterations in the left lower limb. Consequently, a follow-up appointment was scheduled involving the father and the patient’s brother.

**Figure 2:**
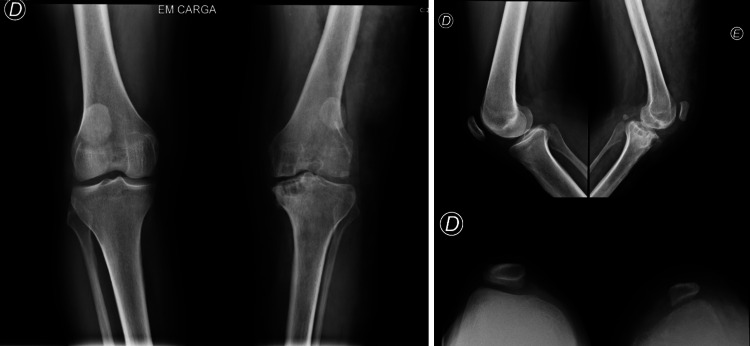
X-rays of the patient’s father’s knees - bilateral patellar hypoplasia associated with osteoarthritis, predominantly on the left side.

The father's medical history included a personal delay in speech development, along with bilateral patellar hypoplasia, knee recurvatum of 15 degrees, a widened gap between the first and second toes, and evident pes planus with pressure callus on the sole on physical examination (Figure 2).

**Figure 3:**
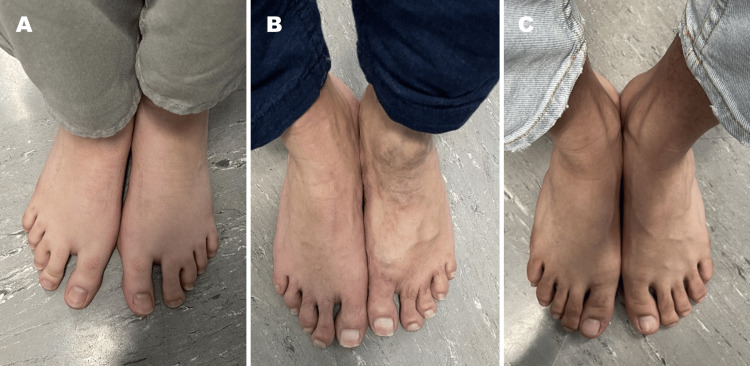
Increased interdigital space between the first and second toes in the patient (A) and patient’s father (B) and normal gap in the patient’s brother (C).

Assessment of the patient’s brother's clinical history and physical examination indicated a delay in speech development without evident signs or symptoms of musculoskeletal disorders (Figure 3).

**Figure 4:**
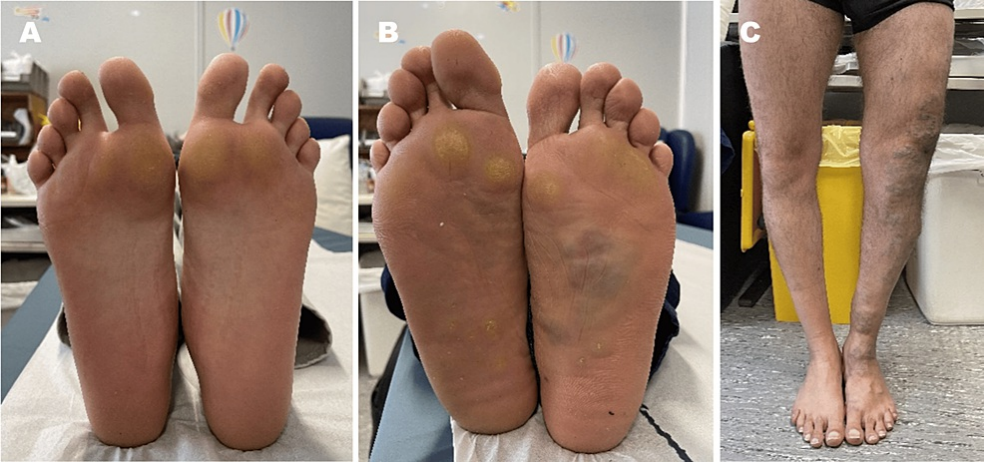
Pressure callus on the sole in the patient (A) and patient’s father (B). Venous alterations in the left lower limb of the patient’s father (C).

No musculoskeletal abnormalities, including elbow deformities, radial head subluxation, metacarpal hypoplasia, winged scapula, scoliosis, or nail dysplasia, as well as facial dysmorphism, sparse scalp hair, or changes in cardiovascular, neurological, renal, or ocular function, were observed in any family member (Figure 4).

## Discussion

The patient we described presents a clinical and radiological picture compatible with Scott-Taor syndrome [3,4], and, as previously referenced, the diagnosis of this syndrome relies on clinical and radiographic findings [4]. As this is an autosomal dominant disease [2], after evaluating the patient's father and brother, we can assume that the patient inherited the condition from her father, while the brother did not exhibit the pathology. Unfortunately, because of logistical constraints, it was not possible to conduct a study involving the patient's mother. While the diagnosis of this condition currently does not offer any targeted treatment, it allows for a better understanding of the clinical presentation described by our patients and integrates this disease as a differential diagnosis, especially from familial patella aplasia-hypoplasia syndrome [5] and nail-patella syndrome [6].

## Conclusions

Scott-Taor syndrome is a rare and benign autosomal dominant disease. Its manifestations can range from knee symptoms such as pain, a sensation of dislocation, or early-onset osteoarthritis associated with patellar a/hypoplasia to a reduced longitudinal arch of the foot along with an increased space between the first and second toe. Additionally, because of its rarity, there are very few studies concerning this pathology. For these reasons, considering this diagnosis is crucial for a better understanding of the pathology and to effectively contextualize the patient's clinical findings.
